# Impact on visual acuity and psychological outcomes of ranibizumab and subsequent treatment for diabetic macular oedema in Japan (MERCURY)

**DOI:** 10.1007/s00417-021-05308-8

**Published:** 2021-09-03

**Authors:** Taiji Sakamoto, Masahiko Shimura, Shigehiko Kitano, Masahito Ohji, Yuichiro Ogura, Hidetoshi Yamashita, Makoto Suzaki, Kimie Mori, Yohei Ohashi, Poh Sin Yap, Takeumi Kaneko, Tatsuro Ishibashi, for the MERCURY Study Group

**Affiliations:** 1 Department of Ophthalmology, Kagoshima University, 8-35-1 Sakuragaoka, Kagoshima, 890-8544 Japan; 2 Department of Ophthalmology, Tokyo Medical University Hachioji Medical Center, Tokyo, Japan; 3 Diabetes Center, Tokyo Women’s Medical University, Tokyo, Japan; 4 Department of Ophthalmology, Shiga University of Medical Science, Otsu, Shiga Japan; 5 Department of Ophthalmology and Visual Science, Nagoya City University, Nagoya, Aichi Japan; 6 Department of Ophthalmology and Visual Sciences, Yamagata University, Yamagata, Japan; 7 Medical Division, Novartis Pharma K.K., Tokyo, Japan; 8 Novartis Corporation (M) Sdn. Bhd., Selangor, Malaysia; 9 Department of Ophthalmology, Kyushu University, Fukuoka, Japan

**Keywords:** Anti-VEGF, Diabetic macular oedema, HADS, Ranibizumab, Real-world data, Visual acuity

## Abstract

**Purpose:**

The MERCURY study aimed to evaluate the effects on visual acuity and psychological symptoms, and safety, of ranibizumab and subsequent treatment in patients with diabetic macular oedema (DME) and impaired visual acuity (VA). We report data from the prespecified 12-month interim analysis.

**Methods:**

This was a 24-month, phase 4, open-label, single-arm, prospective, observational study conducted at 20 specialised retinal centres in Japan. Participants were 209 patients with DME and impaired VA, not previously treated with either intravitreal or systemic anti-vascular endothelial growth factor (anti-VEGF) agents, who initiated ranibizumab 0.5 mg per investigator discretion. Following ranibizumab administration, patients were treated per routine clinical practice. Other treatments were allowed. The main outcome measure was the mean change in best-corrected VA (BCVA) in logarithmic minimum angle of resolution (logMAR) from baseline to month 12. An exploratory objective was to assess patients’ psychological status using the Hospital Anxiety and Depression Scale (HADS).

**Results:**

The mean ± standard deviation BCVA at baseline was 0.43 ± 0.39 logMAR. The mean number of injections of ranibizumab and anti-VEGF agents from baseline to month 11 was 3.2 ± 2.0 and 3.6 ± 2.4, respectively. The BCVA change from baseline to 12 months was − 0.08 ± 0.34 logMAR (*p* = 0.011), showing a significant improvement; the HADS-anxiety score also decreased significantly (*p* = 0.001) and the depression score decreased numerically (*p* = 0.080).

**Conclusion:**

MERCURY study data confirm the effectiveness of real-world treatment initiated with ranibizumab in Japanese patients with DME. In addition, treatment was able to positively influence anxiety via VA improvement.

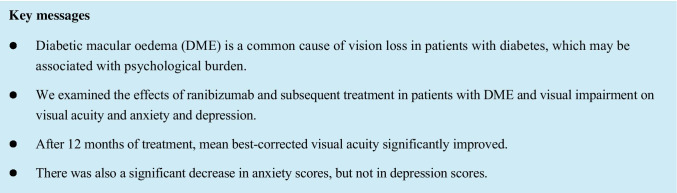

**Supplementary Information:**

The online version contains supplementary material available at 10.1007/s00417-021-05308-8.

## Introduction

Diabetic macular oedema (DME) is the most common cause of vision loss in patients with diabetes [1]. Estimates in 2017 suggested that 451 million people globally are affected by diabetes [2], and up to 15.3% of them have DME [3]. As the prevalence of diabetes continues to increase [2], the burden of DME is also expected to grow. The current mainstay of treatment for DME is anti-vascular endothelial growth factor (VEGF) therapy [4–6].

Like other chronic health conditions [7], diabetes is known to be associated with elevated levels of depression and anxiety [8]. In a cross-sectional study of 2,049 patients with diabetes, investigators found evidence for high levels of anxiety and depression (defined as a score ≥ 8 on the Hospital Anxiety and Depression Scale [HADS]), compared with general population samples [9]. Several subsequent publications have confirmed that HADS scores are higher in patients with diabetic complications than in those without [9–11]. Moreover, studies have indicated that the severity of diabetic retinopathy and/or DME may be associated with poor psychosocial functioning [12] and an elevated level of depression [11]. Poor visual acuity (VA) in the better eye (BE) has also been linked with anxiety and/or depression in other diseases of the eye, including age-related macular degeneration [13, 14] and glaucoma [15]. This is consistent with data which indicate that, in older adults, visual impairment in the BE is associated with a high prevalence of anxiety and depression (measured using HADS and other psychological assessments), compared with normally-sighted peers [16].

It has been demonstrated that it is possible to improve HADS-anxiety (HADS-A) and HADS-depression (HADS-D) scores in visually impaired individuals by correcting low vision [17]; however, to date, there have been few reports evaluating the influence of DME treatment on mental status in patients with DME and impaired VA. Moreover, to our knowledge, there have been no studies to assess the psychological impact of DME treatment in patients using a specific psychological screening tool, such as HADS; indeed, other researchers have noted that this is an area of research that would benefit from additional investigation [12].

Ranibizumab, the first approved anti-VEGF agent for DME, is a recombinant humanised monoclonal antibody fragment that binds to all isoforms of VEGF-A [18]. It is administered intravitreally at a dose of 0.5 mg (Europe and Japan [19]) or 0.3 mg (USA) for DME. Phase 3 clinical studies have shown that intravitreal ranibizumab, either as monotherapy or combined with laser treatment, was more effective than laser alone for the treatment of DME over ≥ 1 year [20, 21].

DME is a long-term condition that places a considerable burden on patients and raises several questions regarding optimal treatment. Repeated intravitreal injections are generally required to maintain improved VA in DME patients [22]. However, the standard criteria for defining stability of VA and disease activity, including guidance for the timing of initiation, interruption and retreatment of ocular anti-VEGF agents, are still not well characterised.

The MERCURY study, a prospective, observational study, was designed to evaluate the effectiveness in terms of VA and safety of ranibizumab and subsequent therapy in the real-world clinical setting for patients with DME who had impaired VA. Additionally, the study aimed to assess the psychological impact associated with the expected VA improvement following anti-VEGF treatment in patients with DME.

## Methods

### Study design

This was a 24-month, phase 4, open-label, single-arm, multicentre, prospective, observational study in DME patients with impaired VA in Japan (Online Resource 2; Online Resource 3). The MERCURY study (JapicCTI-173610) has enrolled patients with DME who initiated ranibizumab 0.5 mg in daily clinical practice. This report includes data (including the primary outcome measure) from the prespecified 12-month interim analysis (cut-off date: September 9, 2019).

All participating patients were to receive at least one ranibizumab injection, but this was an observational study to assess safety and effectiveness in routine clinical practice; thus, other treatments were allowed.

The study protocol and informed consent form were reviewed and approved by each centre’s institutional review board (Online Resource 4), and all patients or their legal representatives provided written informed consent. The study was conducted in accordance with the principles of the Declaration of Helsinki and the guidelines for Good Clinical Practice, Good Post-marketing Study Practice and Good Pharmacoepidemiology Practices. A detailed study design is provided in Online Resource 3.

### Patients

Patients eligible for inclusion were those with DME who had impaired VA as judged by the investigator; aged ≥ 20 years; initiating treatment with ranibizumab for the first time and not previously treated with either intravitreal or systemic anti-VEGF agents; and who anticipated being able to visit the study centre for at least 1 year. Exclusion criteria were simultaneous participation in other investigational studies; planned treatment with systemic anti-VEGF agents within 1 year from baseline; contraindication or hypersensitivity to ranibizumab or its excipients; or diagnosed or suspected infection, or inflammation, in and/or around the eye.

### Treatment

The only recommendation regarding dose, frequency or duration of treatment was that patients received ranibizumab according to the Japanese package insert [19]. Ranibizumab (0.5 mg) was administered pro re nata (PRN) by intravitreal injection (0.05 mL) on a monthly basis. From the second injection (month 1), other ocular anti-VEGF agents were allowed at the investigator’s discretion. Additional adjunctive treatments (e.g. photocoagulation, intraocular injection of steroid or vitrectomy) were not restricted during the study period.

Patients were able to receive ranibizumab injections in one or both eyes. The first eye receiving a ranibizumab injection was considered the primary treated eye (PTE). If the second eye also received a ranibizumab injection prior to other anti-VEGF agent injections, it was considered the secondary treated eye (STE). If both eyes were treated on the same date, the eye with the earliest diagnosis date was considered the PTE. If both eyes had the same diagnosis date, one eye was chosen as the PTE by the investigator.

### Study outcomes and measures

The primary study objective was to describe the effectiveness of ranibizumab and subsequent treatment in DME patients with impaired VA in clinical settings. This was evaluated by assessing mean change in best-corrected VA (BCVA) in logarithmic minimum angle of resolution (logMAR) from baseline to month 12.

Secondary objectives were to characterise the effectiveness of ranibizumab and subsequent treatment by evaluating the monthly changes in BCVA in logMAR and central subfield thickness (CSFT) measured by optical coherence tomography over 12 months. A further secondary objective was to characterise safety during the study.

Exploratory objectives were to assess the psychological status of patients using the HADS [23]. The HADS (Japanese translation [24]) was measured at baseline, month 3 and month 12. The HADS is a self-rated questionnaire and consists of seven items for anxiety (HADS-A) and seven items for depression (HADS-D), with a score of 0 to 3 for each. The resulting total score ranges from 0 to 21 for each subscale, with higher scores indicating more severe symptoms; a score of ≥ 8 indicates subthreshold anxiety or depression. Detailed study outcomes and measures are outlined in Online Resource 3.

### Statistical methods

The planned study size was 200 patients based on prior studies [20, 21]. Further details of sample size calculation, summary statistics and analysis set definitions are outlined in Online Resource 3. Briefly, data were analysed descriptively and summarised together with estimates and corresponding 95% confidence intervals (CIs) as appropriate. The paired *t*-test was performed to evaluate mean changes and the chi-square test used to compare categorical variables. Pearson’s correlation coefficient was used to assess correlations between continuous variables. A *p* value of < 0.05 was considered statistically significant.

## Results

### Patients

In total, 209 patients were enrolled; of these, 192 (91.9%) completed 12 months of observation. The most common reason for discontinuation was a withdrawal of consent (*n* = 11 [5.3%]); no patients discontinued the study due to adverse events (AEs). Full details of patient disposition are shown in Online Resource 5. The safety set comprised all 209 enrolled patients. For effectiveness analyses, the PTE set also contained 209 patients (100%) and the STE set contained 61 patients (29.2%).

Table 1 shows the baseline characteristics of the PTE set. The mean age of patients was 64.4 years and 129 (61.7%) were males. One-third of patients had proliferative diabetic retinopathy (*n* = 68; 32.5%). Just over half of patients (*n* = 120; 57.4%) had undergone previous DME treatment prior to this study, including panretinal photocoagulation (*n* = 69), ocular steroid injection (*n* = 48), and laser photocoagulation (*n* = 36). The mean baseline BCVA (logMAR) was 0.43 in the PTE set (equivalent to 63.5 early treatment diabetic retinopathy study [ETDRS] letters). Relevant non-ocular disease characteristics such as glycated haemoglobin (HbA1c), blood pressure and medical history are presented in Online Resource 6.

**Table 1:** Baseline patient demographics, and disease and ocular characteristics (PTE set)

Variable	PTE, *N* = 209
Age (years), mean ± SD	64.4 ± 12.8
Sex, *n* (%)	
Male	129 (61.7)
Female	80 (38.3)
Diabetes type, *n* (%)	
Type I	4 (1.9)
Type II	205 (98.1)
Time since first diagnosis of diabetes (years), mean ± SD	13.0 ± 11.1
BCVA (logMAR), mean ± SD	0.43 ± 0.39
CSFT (µm), *n*	203
Mean ± SD	459.0 ± 138.7
Time from DME diagnosis (years), *n*	170
Mean ± SD	0.79 ± 1.74
Type of DME, *n*	204
Unilateral, *n* (%)	82 (39.2)
Bilateral, *n* (%)	122 (58.4)
Lens status (phakic), *n* (%)	125 (59.8)
Classification of diabetic retinopathy, *n* (%)	
Mild NPDR	20 (9.6)
Moderate NPDR	56 (26.8)
Severe NPDR	59 (28.2)
PDR	68 (32.5)
Prior DME treatment, *n* (%)	
Any DME treatment	120 (57.4)
Grid/focal laser photocoagulation	36 (17.2)
Intravitreal/subtenon steroid injection	48 (23.0)
Vitrectomy	5 (2.4)
Panretinal photocoagulation	69 (33.0)
Other	10 (4.8)

BCVA, best-corrected visual acuity; CSFT, central subfield thickness; DME, diabetic macular oedema; logMAR, logarithm of the minimum angle of resolution; NPDR, non-proliferative diabetic retinopathy; PDR, proliferative diabetic retinopathy; PTE, primary treated eye; SD, standard deviation

### Effectiveness outcomes

**Fig. 1:**
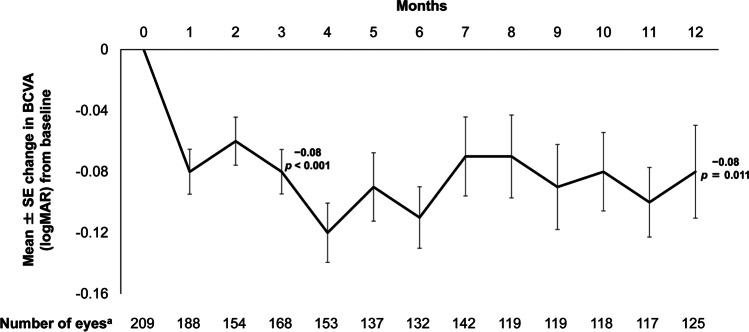
Mean ± SE change in BCVA (logMAR) over time (PTE set). *p* values were calculated using a 1-sample paired *t*-test versus baseline values. ^a^The number of eyes at each month corresponds to the number of patients who attended the study visit and were measured for visual acuity at the corresponding month. BCVA, best-corrected visual acuity; logMAR, logarithm of the minimum angle of resolution; PTE, primary treated eye; SE, standard error

The results for mean change in BCVA from baseline to month 12 for the PTE set are shown in Fig. 1. The BCVA value (± standard deviation [SD]) at baseline was 0.43 ± 0.39 logMAR. Significant improvements in BCVA were shown after 3 months and after 12 months, as shown by the change in BCVA from baseline to 3 months (− 0.08 ± 0.19 logMAR, *p* < 0.001) and from baseline to 12 months (− 0.08 ± 0.34 logMAR, *p* = 0.011). The proportions of patients achieving BCVA improvements of ≤  − 0.1, ≤  − 0.2 and ≤  − 0.3 logMAR units, and the proportion experiencing BCVA deterioration of ≥ 0.3 logMAR units from baseline to month 12 are shown in Online Resource 7.

The mean change ± standard error in CSFT from baseline to month 12 for the PTE set is shown in Online Resource 8. Significant differences from baseline were recorded after 3 months (− 81.5 μm ± 149.0; *p* < 0.001) and 12 months (− 100.3 μm ± 145.1; *p* < 0.001).

### Treatment exposure

The number and frequency of anti-VEGF injections in the PTE and STE sets are shown in Online Resource 9. Overall, the mean ± SD number of injections of ranibizumab and anti-VEGF agents (including ranibizumab) from baseline to month 11 was 3.2 ± 2.0 and 3.6 ± 2.4, respectively, in the PTE set. There were 91 injections of anti-VEGF agents other than ranibizumab administered in the PTE set; almost all (*n* = 90) were aflibercept, and one was bevacizumab. The number of eyes in the PTE set with adjunctive therapy during the same period was 88 (42.1%).

### Visual acuity outcomes and treatment frequency or systemic factor

**Fig. 2:**
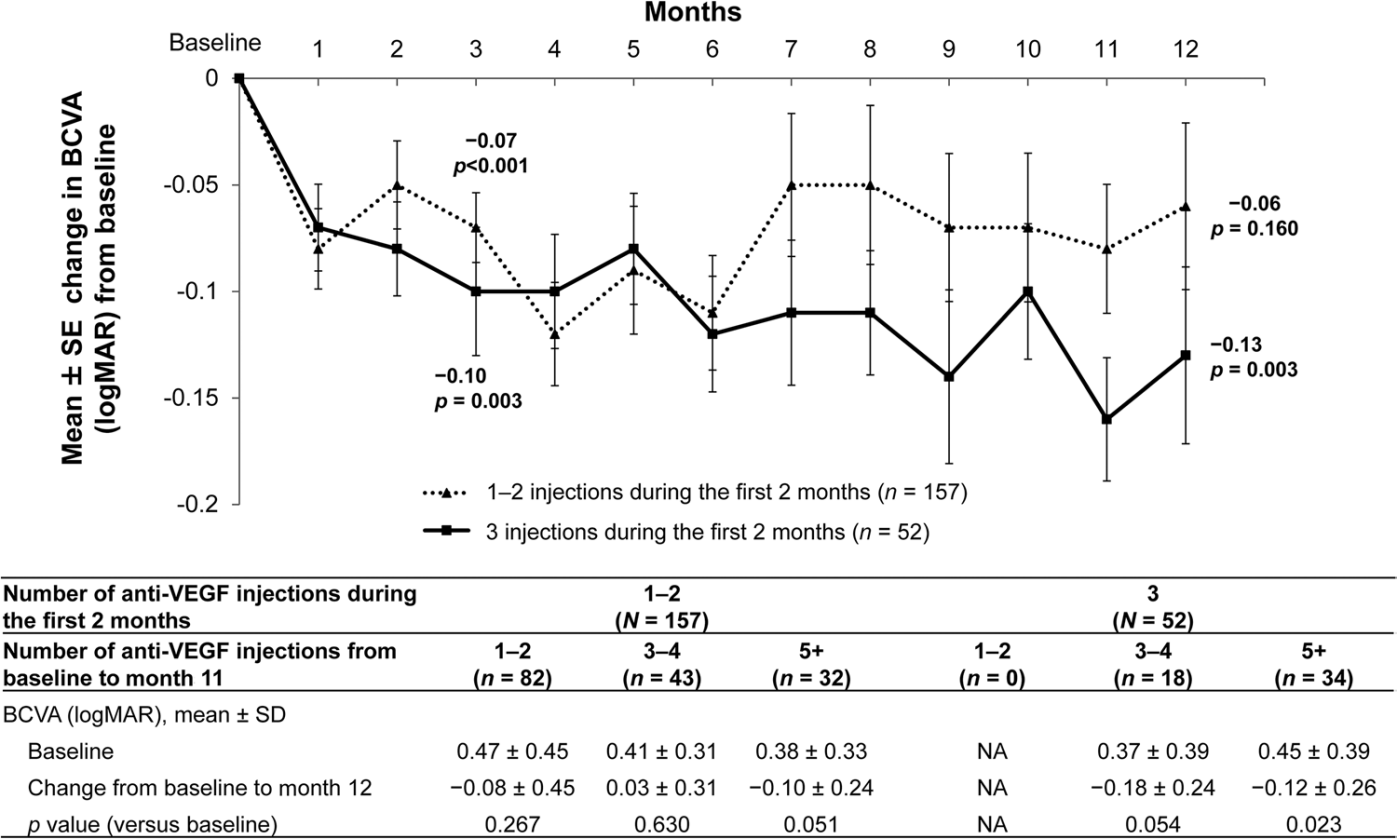
Mean change in BCVA (logMAR) from baseline to month 12 according to the number of anti-VEGF injections administered during the first 2 months (PTE set). Mean BCVA (logMAR) values at baseline were 0.43 and 0.42 in patients who received 1–2 injections from baseline to month 2 and in patients who received 3 injections from baseline to month 2, respectively. In patients who received 1–2 injections from baseline to month 2, the mean number of total injections was 3.0. In patients who received 3 injections from baseline to month 2, the mean number of total injections was 5.7. *p* values were calculated using the paired *t*-test versus baseline values. BCVA, best-corrected visual acuity; logMAR, logarithm of the minimum angle of resolution; NA, not applicable; PTE, primary treated eye; SD, standard deviation; SE, standard error; VEGF, vascular endothelial growth factor

In the PTE group, patients who received three anti-VEGF injections during the first 2 months demonstrated better BCVA improvement compared with those who received one or two injections during the same period (Fig. 2). This improvement was irrespective of the total number of injections administered during the study.

### Exploratory outcomes

**Fig. 3:**
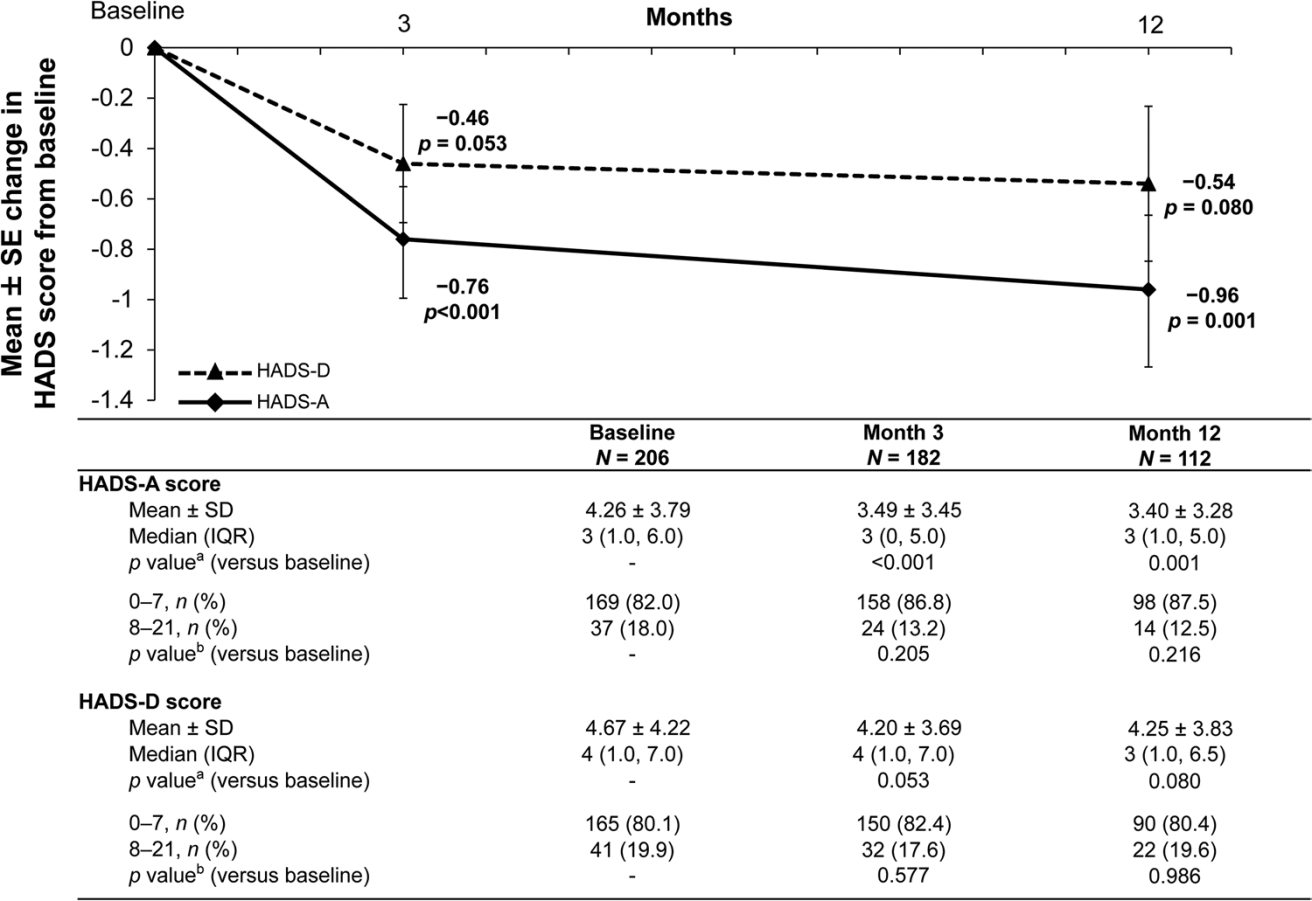
Mean change in HADS scores from baseline (safety set). ^a^*p* values were calculated using the paired *t*-test versus baseline values. ^b^*p* values were calculated using the chi-square test versus baseline values. HADS, Hospital Anxiety and Depression Scale; HADS-A, HADS anxiety subscale; HADS-D, HADS depression subscale; IQR, interquartile range; SD, standard deviation; SE, standard error

The mean changes in HADS score from baseline to months 3 and 12, and the averaged values over time, are described in Fig. 3. The mean ± SD baseline HADS-A and HADS-D scores were 4.26 ± 3.79 and 4.67 ± 4.22, respectively. At month 3, the HADS-A score decreased significantly by − 0.76 ± 2.81 (*p* < 0.001), with the decrease continuing at month 12 (− 0.96 ± 3.13; *p* = 0.001). At months 3 and 12, the HADS-D score decreased by − 0.46 ± 3.16 (*p* = 0.053) and − 0.54 ± 3.26 (*p* = 0.080), respectively.

Overall, 37/206 (18.0%) and 41/206 (19.9%) cases had subthreshold anxiety and depression (scores of ≥ 8), respectively. There was a downward trend in anxiety over time, with subthreshold anxiety present in 24/182 (13.2%) and 14/112 (12.5%) patients at months 3 and 12, respectively. Subthreshold depression did not notably alter following treatment; 32/182 (17.6%) and 22/112 (19.6%) patients had subthreshold depression at months 3 and 12, respectively.

To investigate the impact of DME treatment on HADS-A scores, we evaluated the relationship between HADS and BCVA of the BE (BE BCVA) and between HADS and the number of anti-VEGF injections. At baseline, of the 209 eyes in the PTE set, 44 (21.1%) were classed as the BE, 131 (62.7%) as the worse eye and 25 (12.0%) as having equivalent vision with the other eye; data for nine eyes (4.3%) were missing. In the STE set, the numbers were 35 (57.4%), 14 (23.0%) and 12 (19.7%), respectively; no data were missing for this group. Mean BCVA in the BE at baseline was 0.21 ± 0.34 logMAR, and in the worse eye was 0.52 ± 0.45 logMAR (Online Resource 10). After 12 months of treatment to both eyes, BE BCVA improved significantly from baseline (− 0.06 ± 0.26 logMAR; *p* = 0.013). Changes in the HADS-A score were evaluated according to BE BCVA changes (improved or maintained/deteriorated). HADS-A scores significantly decreased from baseline to month 12 in the improved group (− 1.35 ± 3.43; *p* = 0.008) but not in the maintained/deteriorated group (− 0.53 ± 2.85; *p* = 0.191) (Table 2). An additional subgroup analysis was conducted for patients who received DME treatment only for the PTE during the first year (*n* = 62). The results demonstrated a similar trend to those of the overall PTE set, with a significant decrease in the HADS-A scores in the improved group (− 1.56 ± 3.36; *p* = 0.023) but not in the maintained/worsened group (− 0.97 ± 3.08; *p* = 0.096). There was no correlation between the number of anti-VEGF injections for both eyes from baseline to month 11 and changes in the HADS-A score from baseline to month 12 (*r* = 0.027; *p* = 0.778) (Online Resource 11).

**Table 2:** HADS change from baseline to month 12 according to BE BCVA (logMAR) change (safety set)

HADS score	Improved group^a^, *n* = 54	Maintained/deteriorated group^b^, *n* = 53	*p* value (between group)
HADS-A			
Baseline, *n*	54	53	
Mean ± SD	4.41 ± 3.86	4.36 ± 3.80	
Change from baseline to month 12, *n*^*c*^	49	51	
Mean ± SD	− 1.35 ± 3.43	− 0.53 ± 2.85	0.197
95% CI	(− 2.33, − 0.36)	(− 1.33, 0.27)	
* p* value (vs baseline)	0.008	0.191	
HADS-D			
Baseline, *n*	54	53	
Mean ± SD	4.56 ± 4.05	4.72 ± 4.06	
Change from baseline to month 12, *n*^*c*^	49	51	
Mean ± SD	− 0.90 ± 3.37	− 0.45 ± 3.36	0.508
95% CI	(− 1.87, 0.07)	(− 1.40, 0.49)	
* p* value (vs baseline)	0.068	0.343	

^a^Improved = BE BCVA (logMAR) change from baseline to month 12 was < 0

^b^Maintained/deteriorated = BE BCVA (logMAR) change from baseline to month 12 was ≥ 0

^c^Patients with evaluable data at baseline and month 12

Nominal *p* values (vs baseline) were calculated using the paired *t*-test. Nominal *p* values between the groups were calculated using a 2-sample *t*-test

The eye with better BCVA (higher decimal or lower logMAR) compared with the opposite eye was considered as the ‘better eye’

BE BCVA, better eye best-corrected visual acuity; CI, confidence interval; HADS, Hospital Anxiety and Depression Scale; HADS-A, HADS anxiety subscale; HADS-D, HADS depression subscale; logMAR, logarithm of the minimum angle of resolution; SD, standard deviation

### Safety

The safety data are summarised in Online Resource 12. Overall, 19 patients (9.1%) in the safety set reported ocular serious AEs (SAEs), of which one event of vitreous haemorrhage was suspected to be related to ranibizumab. In contrast, 29 patients (13.9%) reported non-ocular SAEs, of which one event of cerebral infarction was suspected to be related to ranibizumab. Three deaths were reported during the study and were caused by cardiac failure, suicidal behaviour and myocardial infarction; none was suspected to be related to ranibizumab. Details of ocular and non-ocular SAEs are provided in Online Resource 13.

## Discussion

These results from the MERCURY study, a real-world, observational analysis of Japanese patients with DME and impaired VA, show that 12 months after initiation of ranibizumab treatment, mean BCVA (logMAR) values were significantly improved (*p* = 0.011), as was CSFT (*p* < 0.001). Over the same duration, the HADS-A score decreased significantly (*p* = 0.001) and the HADS-D score decreased numerically (*p* = 0.080). To the best of our knowledge, this is the first study to assess the psychological effects of anti-VEGF treatments for DME patients using the HADS, a specific psychological measure.

The mean BCVA gain at month 12 in MERCURY (mean change − 0.08 logMAR, equivalent to + 4 ETDRS letters) is comparable with results from other real-world studies (Online Resource 14). In two retrospective studies of 12 months’ duration, the mean VA gains were + 6.6 letters [25] and + 4.7 [26] letters with a mean of 7.2 and 3.1 injections, respectively. In the prospective real-world OCEAN [27], BOREAL-DME [28] and LUMINOUS [29] studies, the mean VA gains during 12 months were + 4 letters, + 7.4 letters and + 3.5 letters, achieved with a mean of 4.4, 5.1 and 4.5 injections, respectively. Moreover, other studies with longer duration have reported improvements of + 6.6 letters with a mean of 7.7 injections over 4 years [30] and + 2 letters with a mean of 3.8 injections over 2 years [31].

Conversely, we can see that the number of injections and the BCVA gains observed in MERCURY and in other real-world studies was slightly lower compared with the results reported from randomised, controlled trials [20, 21]. This is consistent with a previous report which suggested that use of ranibizumab in routine clinical practice resulted in less frequent injections and reduced effectiveness compared with the drug registration trials [32]. It is difficult to achieve adequate treatment with anti-VEGF agents in real-world settings, and one reason for this appears to be the cost of therapy. Based on the results of a questionnaire evaluating anti-VEGF therapy, 85.8% of physicians responded that financial burden was an important factor which influenced the rate of continuation of injections [33]. Additionally, the fact that patients in MERCURY had relatively high baseline BCVA (Online Resource 14) and well-managed diabetic status (i.e. HbA1c; Online Resource 6) may have resulted in less frequent injections and comparative undertreatment in this study in relation to other trials. Interestingly, our study showed that patients who had three injections during the first 2 months had improved BCVA compared with those who did not. This improvement was observed irrespective of the overall number of injections from baseline to month 11. This regimen, of three injections during the first 2 months, has previously been shown to provide benefit to patients [29] and might be an alternative (and less costly) solution for DME patients in real-world settings.

Previous studies have suggested that the prevalences of anxiety and depression are higher in older patients with poor VA [16, 34], in patients with diabetes [10] and in those with diabetic ocular complications [11, 12, 35]. Of note, Rees et al. [11] reported that 24.3% and 16.3% of patients with DME, respectively, had HADS-A and HADS-D scores of ≥ 8. This is comparable to our results showing that 18.0% and 19.9%, respectively, had HADS-A and HADS-D scores of ≥ 8. However, our results also showed that the HADS-A score decreased significantly (*p* = 0.001) and the HADS-D score decreased numerically (*p* = 0.080) following DME treatment. Furthermore, a downward trend in the number of patients with subthreshold anxiety was also observed during the study. Visual impairment in the BE is known to increase the prevalence of subthreshold anxiety and correlate with increased HADS-A scores [14, 16].

In our stratified analysis of BE BCVA, the HADS-A score significantly decreased in the improved group, but not in the maintained/deteriorated group. This suggests that improvement in BE BCVA following DME treatment was able to lessen the anxiety symptoms felt by the patients in our study. Additionally, the results from the subgroup analysis of patients who received DME treatment only for PTE was consistent with the data from the overall patient population. That the same trend was observed in the subgroup supports the premise that improvement in BE BCVA following DME treatment is associated with a lessening of anxiety symptoms. However, there was no significant correlation between the HADS-A score and the number of anti-VEGF injections. We consider that there is a low possibility that changes in systemic factors of patients affected the HADS scores during our study, based on a previous report that the values of systemic factors, such as blood glucose, blood pressure and lipids, were unrelated to the HADS-A score in patients with diabetic retinopathy [11]. Thus, DME treatment initiated with ranibizumab may provide additional benefit to patients with DME in the real-world setting by diminishing psychological symptoms via the improvement of VA.

Notably, the reason for the differing results according to HADS-A and HADS-D in the study was unclear. It has been reported that deteriorated BE BCVA worsens the scores of both HADS subscales [14, 34], but there is a lack of detailed clinical data, and further investigations are needed to clarify this point. In our study, there was no change in HADS-D scores in patients with DME after 1 year of treatment, and this result is in line with the previous study by Rees et al. [11], in which no correlation between DME and HADS-D was reported.

### Limitations

The potential limitations of the study are primarily due to its observational study design, but it must be remembered that this type of study is best suited to obtain real-life data. Although the use of the Landolt C chart was recommended to assess BCVA, the investigators were allowed to measure vision per their usual practice; thus, there may have been variability in the quality of visual acuity measurements. The lack of a comparator arm means that the effectiveness and safety of ranibizumab treatment cannot be directly compared with other studies of interventional therapy. Moreover, as with all measures relying on subjective patient responses, the use of psychological questionnaires such as HADS presents some difficulties in proving statistically significant changes in mental symptoms by treatment or between groups. Finally, the variable visit schedule of each patient means that there was a much lower rate of data available for evaluation at month 12 (as described in the footnotes of each figure) than the discontinuation rate would suggest. Most patients visited the clinic and underwent evaluation every few months. However, since the fluctuation of BCVA change at each month was small, we consider the results from the patients assessed at month 12 to be indicative of the overall population.

## Conclusions

Data from the open-label, observational MERCURY study confirm the effectiveness and safety of DME treatment initiated with ranibizumab in Japanese patients. In addition, treatment was able to provide potential positive effects on anxiety via VA improvement.

## Supplementary Information

Below is the link to the electronic supplementary material.

Supplementary file1 (PDF 146 KB)

Supplementary file2 (PDF 118 KB)

Supplementary file3 (PDF 233 KB)

Supplementary file4 (PDF 132 KB)

Supplementary file5 (PDF 186 KB)

Supplementary file6 (PDF 165 KB)

Supplementary file7 (PDF 202 KB)

Supplementary file8 (PDF 267 KB)

Supplementary file9 (PDF 206 KB)

Supplementary file10 (PDF 245 KB)

Supplementary file11 (PDF 299 KB)

Supplementary file12 (PDF 165 KB)

Supplementary file13 (PDF 135 KB)

Supplementary file14 (PDF 207 KB)

## Data Availability

The data relevant to the study findings are included in the article or uploaded as supplementary information. No additional data are available.
