# Supplementary material for: Impact on visual acuity and psychological outcomes of ranibizumab and subsequent treatment for diabetic macular oedema in Japan (MERCURY)
Source: Graefes Arch Clin Exp Ophthalmol. 2021 Sep 3;260(2):477–87. doi: 10.1007/s00417-021-05308-8 (PMC8786783; doi:10.1007/s00417-021-05308-8)
Supplement: Supplementary file 1 — Supplementary file1 (PDF 146 KB) [file 417_2021_5308_MOESM1_ESM.pdf]

Graefe's Archive for Clinical and Experimental Ophthalmology

Impact on visual acuity and psychological outcomes of ranibizumab and subsequent treatment for diabetic macular oedema in Japan (MERCURY)

Taiji Sakamoto, Masahiko Shimura, Shigehiko Kitano, Masahito Ohji, Yuichiro Ogura, Hidetoshi Yamashita, Makoto Suzaki, Kimie Mori, Yohei Ohashi, Poh Sin Yap, Takeumi Kaneko, Tatsuro Ishibashi, for the MERCURY Study Group

Corresponding author:

Taiji Sakamoto

Department of Ophthalmology, Kagoshima University, 8-35-1 Sakuragaoka, Kagoshima 890-8544, Japan

Tel: +81 99-275-5402

Fax: +81 99-265-4894

Email: tsakamot@m3.kufm.kagoshima-u.ac.jp

Online Resource 1. The MERCURY study investigators and advisors

Study sites	Principal investigators
Tokyo Medical University Hachioji Medical Center	Masahiko Shimura
Hyogo Prefectural Amagasaki General Medical Center	Hideyasu Oh
Mie University	Masahiko Sugimoto
Kagoshima University	Taiji Sakamoto
St. Marianna University School of Medicine	Jiro Kogo
St. Marianna University School of Medicine	Hitoshi Takagi
Tokushima University	Yoshinori Mitamura
Sapporo City General Hospital	Hiroko Imaizumi
Kobe University	Sentaro Kusuhara
Nara Medical University	Nahoko Ogata
Tokyo Women's Medical University	Shigehiko Kitano
University of Fukui	Yoshihiro Takamura
Kyoto University	Tomoaki Murakami
National Defense Medical College	Masaru Takeuchi
Shinshu University	Yuichi Toriyama
Kyushu University	Shigeo Yoshida
Kyushu University	Toshio Hisatomi
Kyushu University	Keijiro Ishikawa
Kyorin University	Akito Hirakata
University of Tsukuba	Fumiki Okamoto

Yamagata University	Sachi Abe
Yamagata University	Katsuhiro Nishi
Shiga University of Medical Science	Osamu Sawada
Nagoya City University	Miho Nozaki

**Affiliations**	**Study advisors**
Kagoshima University	Taiji Sakamoto
Kyushu University	Tatsuro Ishibashi
Yamagata University	Hidetoshi Yamashita
Nagoya City University	Yuichiro Ogura
Shiga University of Medical Science	Masahito Ohji
Tokyo Women's Medical University	Shigehiko Kitano
Tokyo Medical University Hachioji Medical Center	Masahiko Shimura
