# Supplementary material for: Impact on visual acuity and psychological outcomes of ranibizumab and subsequent treatment for diabetic macular oedema in Japan (MERCURY)
Source: Graefes Arch Clin Exp Ophthalmol. 2021 Sep 3;260(2):477–87. doi: 10.1007/s00417-021-05308-8 (PMC8786783; doi:10.1007/s00417-021-05308-8)
Supplement: Supplementary file 3 — Supplementary file3 (PDF 233 KB) [file 417_2021_5308_MOESM3_ESM.pdf]

Graefe's Archive for Clinical and Experimental Ophthalmology

Impact on visual acuity and psychological outcomes of ranibizumab and subsequent treatment for diabetic macular oedema in Japan (MERCURY)

Taiji Sakamoto, Masahiko Shimura, Shigehiko Kitano, Masahito Ohji, Yuichiro Ogura, Hidetoshi Yamashita, Makoto Suzaki, Kimie Mori, Yohei Ohashi, Poh Sin Yap, Takeumi Kaneko, Tatsuro Ishibashi, for the MERCURY Study Group

Corresponding author:

Taiji Sakamoto

Department of Ophthalmology, Kagoshima University, 8-35-1 Sakuragaoka, Kagoshima 890-8544, Japan

Tel: +81 99-275-5402

Fax: +81 99-265-4894

Email: tsakamot@m3.kufm.kagoshima-u.ac.jp

Online Resource 3. Supplementary Methodology

Study design

The MERCURY study is being conducted at 20 specialised retinal centres nationwide, and has enrolled patients with diabetic macular oedema (DME) who initiated ranibizumab 0.5 mg in daily clinical practice. The study is ongoing; it commenced in April 2017 and is expected to be completed in December 2020. Patients were enrolled between August 2017 and June 2018.

No visit schedule was imposed, and patients were treated per routine medical practice. Data were recorded at each visit in electronic case report format (eCRF) and were regularly checked by field monitors. It was recommended that data should be captured in the eCRF at every visit or a minimum of every 3 months, and investigators were encouraged to follow-up with patients who had not been seen in the centre for at least 6 months in order to capture data. Patients not seen at least once per year were discontinued from the study.

Study outcomes and measures

For assessment of the primary study objective, it was recommended that best-corrected visual acuity (BCVA) be measured by Landolt C chart in a sitting position at an initial testing distance of 5 m (16.4 feet) at every visit; however, the usual practice of the participating investigator was permitted. For statistical analysis, the decimal BCVA value collected was converted into logarithmic minimum angle of resolution (logMAR) units using the formula: logMAR BCVA = $\log_{10}$ (1/decimal BCVA) = $-\log_{10}$ (decimal BCVA).

Secondary objectives also included assessment of treatment exposure (ranibizumab, anti-vascular endothelial growth factor (VEGF) treatments and DME

adjunctive treatments) during the observation period; the percentages of patients achieving BCVA improvements of ≤ −0.1, ≤ −0.2 and ≤ −0.3 logMAR, and the percentage of patients experiencing a BCVA deterioration of ≥ 0.3 logMAR from baseline to month 12; the relationship between treatment effectiveness and safety and baseline characteristics; and characterisation of patients who initiated ranibizumab based on baseline characteristics. Data to month 12 are reported in the interim analysis. For safety, all undesirable signs, symptoms or medical conditions were collected as adverse events (AEs), and the incidence rate and severity of ocular and/or non-ocular AEs and the relationship to treatment were assessed. AEs were coded using the Medical Dictionary for Regulatory Activities (MedDRA) version 22.0.

For exploratory objectives, the Hospital Anxiety and Depression Scale (HADS) measurements consist of seven items for anxiety (HADS-A) and seven items for depression (HADS-D), with a score of 0 to 3 for each, resulting in a possible total score of 0 to 21. Higher scores indicate more severe symptoms. A score of ≥ 8 indicates subthreshold anxiety or depression. The self-rated HADS questionnaire was completed by patients, with the resulting scores entered into the electronic case report form by the medical personnel at the study site. To investigate the impact of BCVA on each HADS change, HADS changes by subgroups of change in BCVA of the better eye (BE) from baseline to month 12 were evaluated. BCVA of the BE changes were calculated using BCVA values of the BE defined at each timepoint assessed (i.e. not defined at baseline). To investigate the impact of the number of anti-VEGF injections on HADS, correlations between anti-VEGF injection number and HADS change were explored.

Study data collected at baseline included patient demographics, non-ocular and ocular characteristics, and medical history/comorbidities. During the study period, information on anti-VEGF treatments and other DME treatments (number, date and reason for treatment) were collected.

Statistical methods

For sample size calculation, prior studies suggest that a mean BCVA change of 0.1 logMAR units from baseline to month 12 (equivalent to five Early Treatment Diabetic Retinopathy Study letters in BCVA) and 0.1 units of standard deviation can be assumed. A sample size of 180 primary treated eyes (PTEs) would yield a 95% confidence interval (CI) with a half-width of approximately 0.015 units. The overall sample size of 200 PTEs was set with the assumption that 10% of participants would not contribute any data at month 12.

The safety set included all enrolled patients who received ≥ 1 dose of ranibizumab during this study and had ≥ 1 safety assessment after the first treatment. The PTE set included all primary treated eyes; the secondary treated eye (STE) set included all secondary treated eyes, and the fellow eye set included all fellow eyes. For effectiveness analyses, only the PTE and STE sets were evaluated.

All data were analysed descriptively and summarised together with estimates and corresponding 95% CIs as appropriate. The paired *t*-test was performed to evaluate mean changes from baseline to month 3 and month 12, and the chi-square test was used to compare categorical variables at baseline and month 3 or month 12. Pearson's correlation coefficient was used to assess the correlation between continuous variables (i.e. BCVA and HADS scores).

All analyses were conducted based on observed data and no imputation method was applied. All statistical calculations were performed using SAS Version 9.4 (SAS Institute Inc., Cary, NC, USA).
