# Supplementary material for: Impact on visual acuity and psychological outcomes of ranibizumab and subsequent treatment for diabetic macular oedema in Japan (MERCURY)
Source: Graefes Arch Clin Exp Ophthalmol. 2021 Sep 3;260(2):477–87. doi: 10.1007/s00417-021-05308-8 (PMC8786783; doi:10.1007/s00417-021-05308-8)
Supplement: Supplementary file 4 — Supplementary file4 (PDF 132 KB) [file 417_2021_5308_MOESM4_ESM.pdf]

Graefe's Archive for Clinical and Experimental Ophthalmology

Impact on visual acuity and psychological outcomes of ranibizumab and subsequent treatment for diabetic macular oedema in Japan (MERCURY)

Taiji Sakamoto, Masahiko Shimura, Shigehiko Kitano, Masahito Ohji, Yuichiro Ogura, Hidetoshi Yamashita, Makoto Suzaki, Kimie Mori, Yohei Ohashi, Poh Sin Yap, Takeumi Kaneko, Tatsuro Ishibashi, for the MERCURY Study Group

Corresponding author:

Taiji Sakamoto

Department of Ophthalmology, Kagoshima University, 8-35-1 Sakuragaoka, Kagoshima 890-8544, Japan

Tel: +81 99-275-5402

Fax: +81 99-265-4894

Email: tsakamot@m3.kufm.kagoshima-u.ac.jp

Online Resource 4. List of Independent Ethics Committees or Institutional Review Boards by study centre

IEC/IRB	Center	City	Province
Tokyo Medical University Hachioji Medical Center Institutional Review Board	Tokyo Medical University Hachioji Medical Center	Hachioji	Tokyo
Hyogo Prefectural Amagasaki General Medical Center Institutional Review Board	Hyogo Prefectural Amagasaki General Medical Center	Amagasaki	Hyogo
Mie University Hospital Institutional Review Board	Mie University Hospital	Tsu	Mie
Kagoshima University Hospital Institutional Review Board	Kagoshima University Hospital	Kagoshima	Kagoshima
St. Marianna University School of Medicine Hospital Institutional Review Board	St. Marianna University School of Medicine Hospital	Kawasaki	Kanagawa
Tokushima University Hospital Institutional Review Board	Tokushima University Hospital	Tokushima	Tokushima
The Ethical Committee of Sapporo City General Hospital	Sapporo City General Hospital	Sapporo	Hokkaido
Kobe University Hospital Institutional Review Board	Kobe University Hospital	Kobe	Hyogo
Nara Medical University	Nara Medical	Kashihara	Nara

Hospital Institutional Review Board	University Hospital		
Tokyo Women's Medical University Hospital Institutional Review Board	Tokyo Women's Medical University Hospital	Shinjuku	Tokyo
University of Fukui Hospital Institutional Review Board	University of Fukui Hospital	Yoshida	Fukui
Kyoto University Hospital Institutional Review Board	Kyoto University Hospital	Kyoto	Kyoto
National Defense Medical College Hospital Institutional Review Board	National Defense Medical College Hospital	Tokorozawa	Saitama
Shinshu University Hospital Institutional Review Board	Shinshu University Hospital	Matsumoto	Nagano
Kyushu University Hospital Institutional Review Board	Kyushu University Hospital	Fukuoka	Fukuoka
Kyorin University Hospital Institutional Review Board	Kyorin University Hospital	Mitaka	Tokyo
University of Tsukuba Hospital Institutional Review Board	University of Tsukuba Hospital	Tsukuba	Ibaraki
Yamagata University Hospital Institutional Review Board	Yamagata University Hospital	Yamagata	Yamagata
Shiga University of Medical Science Hospital Institutional	Shiga University of Medical Science	Otsu	Shiga

Review Board	Hospital		
Nagoya City University Hospital Institutional Review Board	Nagoya City University Hospital	Nagoya	Aichi

IEC, independent ethics committee; IRB, institutional review board.
