# Supplementary material for: Impact on visual acuity and psychological outcomes of ranibizumab and subsequent treatment for diabetic macular oedema in Japan (MERCURY)
Source: Graefes Arch Clin Exp Ophthalmol. 2021 Sep 3;260(2):477–87. doi: 10.1007/s00417-021-05308-8 (PMC8786783; doi:10.1007/s00417-021-05308-8)
Supplement: Supplementary file 6 — Supplementary file6 (PDF 165 KB) [file 417_2021_5308_MOESM6_ESM.pdf]

Graefe's Archive for Clinical and Experimental Ophthalmology

Impact on visual acuity and psychological outcomes of ranibizumab and subsequent treatment for diabetic macular oedema in Japan (MERCURY)

Taiji Sakamoto, Masahiko Shimura, Shigehiko Kitano, Masahito Ohji, Yuichiro Ogura, Hidetoshi Yamashita, Makoto Suzaki, Kimie Mori, Yohei Ohashi, Poh Sin Yap, Takeumi Kaneko, Tatsuro Ishibashi, for the MERCURY Study Group

Corresponding author:

Taiji Sakamoto

Department of Ophthalmology, Kagoshima University, 8-35-1 Sakuragaoka, Kagoshima 890-8544, Japan

Tel: +81 99-275-5402

Fax: +81 99-265-4894

Email: tsakamot@m3.kufm.kagoshima-u.ac.jp

Online Resource 6. Relevant non-ocular medical status and history at baseline (PTE set)

Variable	PTE<br>***N* = 209**
BMI (kg/m^2), *n*	166
Mean ± SD	24.5 ± 3.6
Blood pressure (mmHg), *n*	180
Systolic, mean ± SD	140.8 ± 20.0
Diastolic, mean ± SD	79.4 ± 12.3
HbA1c (%), *n*	140
Mean ± SD	7.3 ± 1.3
HDL-cholesterol (mg/dL), *n*	101
Mean ± SD	55.2 ± 12.7
LDL-cholesterol (mg/dL), *n*	96
Mean ± SD	113.2 ± 39.8
Triglycerides (mg/dL), *n*	118
Mean ± SD	141.5 ± 78.6
eGFR (mL/min/1.73 m^2), *n*	115
Mean ± SD	61.3 ± 27.1
Insulin use, *n* (%)	45 (21.5)
Smoking history, *n*	208
Never smoker, *n* (%)	96 (45.9)
Past smoker, *n* (%)	68 (32.5)

Current smoker, *n* (%)	44 (21.1)
Medical history/comorbidities, *n* (%)	
Diabetic nephropathy	12 (5.7)
Diabetic neuropathy	2 (1.0)
Dyslipidemia	104 (49.8)
Hypertension	131 (62.7)
Myocardial infarction	11 (5.3)
Stroke	17 (8.1)
Thromboembolic event	8 (3.8)

BMI, body mass index; eGFR, estimated glomerular filtration rate; HbA1c, glycated haemoglobin; HDL, high-density lipoprotein; LDL, low density lipoprotein; PTE, primary treated eye; SD, standard deviation
