# Supplementary material for: Impact on visual acuity and psychological outcomes of ranibizumab and subsequent treatment for diabetic macular oedema in Japan (MERCURY)
Source: Graefes Arch Clin Exp Ophthalmol. 2021 Sep 3;260(2):477–87. doi: 10.1007/s00417-021-05308-8 (PMC8786783; doi:10.1007/s00417-021-05308-8)
Supplement: Supplementary file 7 — Supplementary file7 (PDF 202 KB) [file 417_2021_5308_MOESM7_ESM.pdf]

*Graefe's Archive for Clinical and Experimental Ophthalmology*

# Impact on visual acuity and psychological outcomes of ranibizumab and subsequent treatment for diabetic macular oedema in Japan (MERCURY)

Taiji Sakamoto, Masahiko Shimura, Shigehiko Kitano, Masahito Ohji, Yuichiro Ogura, Hidetoshi Yamashita, Makoto Suzaki, Kimie Mori, Yohei Ohashi, Poh Sin Yap, Takeumi Kaneko, Tatsuro Ishibashi, for the MERCURY Study Group

**Corresponding author:**

Taiji Sakamoto

Department of Ophthalmology, Kagoshima University, 8-35-1 Sakuragaoka, Kagoshima 890-8544, Japan

Tel: +81 99-275-5402

Fax: +81 99-265-4894

Email: tsakamot@m3.kufm.kagoshima-u.ac.jp

**Online Resource 7.** BCVA outcomes at month 12 (PTE set)

| **Change, *n* (%)** | **PTE (*N* = 209)** |
|---|---|
| Change in BCVA (logMAR) from baseline to month 12, *n* | 125 |
| ≤−0.1 (improved) | 57 (45.6) |
| ≤−0.2 (improved) | 25 (20.0) |
| ≤−0.3 (improved) | 16 (12.8) |
| <0.3 (stable or improved) | 117 (93.6) |
| ≥0.3 (deteriorated) | 8 (6.4) |

BCVA, best-corrected visual acuity; logMAR, logarithm of the minimum angle of resolution; PTE, primary treated eye.
