# Supplementary material for: Impact on visual acuity and psychological outcomes of ranibizumab and subsequent treatment for diabetic macular oedema in Japan (MERCURY)
Source: Graefes Arch Clin Exp Ophthalmol. 2021 Sep 3;260(2):477–87. doi: 10.1007/s00417-021-05308-8 (PMC8786783; doi:10.1007/s00417-021-05308-8)
Supplement: Supplementary file 9 — Supplementary file9 (PDF 206 KB) [file 417_2021_5308_MOESM9_ESM.pdf]

Graefe's Archive for Clinical and Experimental Ophthalmology

Impact on visual acuity and psychological outcomes of ranibizumab and subsequent treatment for diabetic macular oedema in Japan (MERCURY)

Taiji Sakamoto, Masahiko Shimura, Shigehiko Kitano, Masahito Ohji, Yuichiro Ogura, Hidetoshi Yamashita, Makoto Suzaki, Kimie Mori, Yohei Ohashi, Poh Sin Yap, Takeumi Kaneko, Tatsuro Ishibashi, for the MERCURY Study Group

Corresponding author:

Taiji Sakamoto

Department of Ophthalmology, Kagoshima University, 8-35-1 Sakuragaoka, Kagoshima 890-8544, Japan

Tel: +81 99-275-5402

Fax: +81 99-265-4894

Email: tsakamot@m3.kufm.kagoshima-u.ac.jp

Online Resource 9. Anti-VEGF treatment and summary of other adjunctive DME treatments from baseline to month 11 (safety set)

	**PTE**<br>***N* = 209**	**STE**<br>***N* = 61**
Number of anti-VEGF injections (including ranibizumab)		
Mean ± SD	3.6 ± 2.4	2.8 ± 1.8
Median (IQR)	3.0 (2.0, 5.0)	2.0 (1.0, 4.0)
Number of ranibizumab injections		
Mean ± SD	3.2 ± 2.0	2.4 ± 1.5
Median (IQR)	3.0 (2.0, 4.0)	2.0 (1.0, 3.0)
Number of eyes with other adjunctive DME treatments, *n* (%)		
Any other DME treatment	88 (42.1)	23 (37.7)
Grid/focal laser photocoagulation	30 (14.4)	7 (11.5)
Intravitreal/subtenon steroid injection	29 (13.9)	6 (9.8)
Vitrectomy	8 (3.8)	4 (6.6)
Panretinal photocoagulation	43 (20.6)	13 (21.3)
Other	8 (3.8)	1 (1.6)

DME, diabetic macular oedema; IQR, interquartile range; PTE, primary treated eye; SD, standard deviation; STE, secondary treated eye; VEGF, vascular endothelial growth factor.
