# Supplementary material for: Impact on visual acuity and psychological outcomes of ranibizumab and subsequent treatment for diabetic macular oedema in Japan (MERCURY)
Source: Graefes Arch Clin Exp Ophthalmol. 2021 Sep 3;260(2):477–87. doi: 10.1007/s00417-021-05308-8 (PMC8786783; doi:10.1007/s00417-021-05308-8)
Supplement: Supplementary file 10 — Supplementary file10 (PDF 245 KB) [file 417_2021_5308_MOESM10_ESM.pdf]

Graefe's Archive for Clinical and Experimental Ophthalmology

Impact on visual acuity and psychological outcomes of ranibizumab and subsequent treatment for diabetic macular oedema in Japan (MERCURY)

Taiji Sakamoto, Masahiko Shimura, Shigehiko Kitano, Masahito Ohji, Yuichiro Ogura, Hidetoshi Yamashita, Makoto Suzaki, Kimie Mori, Yohei Ohashi, Poh Sin Yap, Takeumi Kaneko, Tatsuro Ishibashi, for the MERCURY Study Group

Corresponding author:

Taiji Sakamoto

Department of Ophthalmology, Kagoshima University, 8-35-1 Sakuragaoka, Kagoshima 890-8544, Japan

Tel: +81 99-275-5402

Fax: +81 99-265-4894

Email: tsakamot@m3.kufm.kagoshima-u.ac.jp

Online Resource 10. BCVA outcomes in the better eye and worse eye from baseline to month 12 (safety set)

	n	Mean ± SD	95% CI	*p* value (vs baseline)
Better eye BCVA (logMAR)				
At baseline	200	0.21 ± 0.34		
Change from baseline to month 3	138	−0.05 ± 0.17	−0.08, −0.02	0.001
Change from baseline to month 12	108	−0.06 ± 0.26	−0.11, −0.01	0.013
Worse eye BCVA (logMAR)				
At baseline	200	0.52 ± 0.45		
Change from baseline to month 3	138	−0.09 ± 0.27	−0.13, −0.04	<0.001
Change from baseline to month 12	108	−0.07 ± 0.35	−0.14, −0.01	0.030

Nominal *p* values were calculated using the paired *t*-test.

The eye with better BCVA (higher decimal or lower logMAR) compared with the opposite eye was considered as the ‘better eye’, and vice versa for the ‘worse eye’.

BCVA, best-corrected visual acuity; CI, confidence interval; logMAR, logarithm of the minimum angle of resolution; SD, standard deviation.
