# Supplementary material for: Impact on visual acuity and psychological outcomes of ranibizumab and subsequent treatment for diabetic macular oedema in Japan (MERCURY)
Source: Graefes Arch Clin Exp Ophthalmol. 2021 Sep 3;260(2):477–87. doi: 10.1007/s00417-021-05308-8 (PMC8786783; doi:10.1007/s00417-021-05308-8)
Supplement: Supplementary file 12 — Supplementary file12 (PDF 165 KB) [file 417_2021_5308_MOESM12_ESM.pdf]

Graefe's Archive for Clinical and Experimental Ophthalmology

Impact on visual acuity and psychological outcomes of ranibizumab and subsequent treatment for diabetic macular oedema in Japan (MERCURY)

Taiji Sakamoto, Masahiko Shimura, Shigehiko Kitano, Masahito Ohji, Yuichiro Ogura, Hidetoshi Yamashita, Makoto Suzaki, Kimie Mori, Yohei Ohashi, Poh Sin Yap, Takeumi Kaneko, Tatsuro Ishibashi, for the MERCURY Study Group

Corresponding author:

Taiji Sakamoto

Department of Ophthalmology, Kagoshima University, 8-35-1 Sakuragaoka, Kagoshima 890-8544, Japan

Tel: +81 99-275-5402

Fax: +81 99-265-4894

Email: tsakamot@m3.kufm.kagoshima-u.ac.jp

Online Resource 12. Summary of safety (safety set)

**Events, *n* (%)**	**Safety Set**<br>***N* = 209**
Death	3 (1.4)
AE	
Ocular AE	57 (27.3)
Non-ocular AE	89 (42.6)
SAE	
Ocular SAE	19 (9.1)
Non-ocular SAE	29 (13.9)
AE leading to discontinuation of study drug	
Ocular AE	0
Non-ocular AE	3 (1.4)
SAE leading to discontinuation of study drug	
Ocular SAE	0
Non-ocular SAE	3 (1.4)

AE, adverse event; SAE, serious adverse event.
