# Supplementary material for: Impact on visual acuity and psychological outcomes of ranibizumab and subsequent treatment for diabetic macular oedema in Japan (MERCURY)
Source: Graefes Arch Clin Exp Ophthalmol. 2021 Sep 3;260(2):477–87. doi: 10.1007/s00417-021-05308-8 (PMC8786783; doi:10.1007/s00417-021-05308-8)
Supplement: Supplementary file 13 — Supplementary file13 (PDF 135 KB) [file 417_2021_5308_MOESM13_ESM.pdf]

*Graefe's Archive for Clinical and Experimental Ophthalmology*

# Impact on visual acuity and psychological outcomes of ranibizumab and subsequent treatment for diabetic macular oedema in Japan (MERCURY)

Taiji Sakamoto, Masahiko Shimura, Shigehiko Kitano, Masahito Ohji, Yuichiro Ogura, Hidetoshi Yamashita, Makoto Suzaki, Kimie Mori, Yohei Ohashi, Poh Sin Yap, Takeumi Kaneko, Tatsuro Ishibashi, for the MERCURY Study Group

**Corresponding author:**

Taiji Sakamoto

Department of Ophthalmology, Kagoshima University, 8-35-1 Sakuragaoka, Kagoshima 890-8544, Japan

Tel: +81 99-275-5402

Fax: +81 99-265-4894

Email: tsakamot@m3.kufm.kagoshima-u.ac.jp

**Online Resource 13.** Serious adverse events (safety set)

| **Events, *n* (%)** | ***N* = 209** |
|---|---|
| ***Ocular SAEs*** | |
| Number of patients with at least one ocular SAE | 19 (9.1) |
| Eye disorders | 18 (8.6) |
|     Vitreous haemorrhage | 10 (4.8) |
|     Glaucoma | 2 (1.0) |
|     Vitreous adhesions | 2 (1.0) |
|     Cataract | 1 (0.5) |
|     Diabetic retinopathy | 1 (0.5) |
|     Ocular hypertension | 1 (0.5) |
|     Retinal haemorrhage | 1 (0.5) |
|     Tractional retinal detachment | 1 (0.5) |
|     Macular hole | 1 (0.5) |
| Infections and infestations | 1 (0.5) |
|     Endophthalmitis | 1 (0.5) |
| ***Non-ocular SAEs*** | |
| Number of patients with at least one non-ocular SAE | 29 (13.9) |
| Cardiac disorders | 9 (4.3) |
|     Cardiac failure | 4 (1.9) |
|     Angina pectoris | 1 (0.5) |
|     Atrial flutter | 1 (0.5) |

| | |
|---|---|
| Bradycardia | 1 (0.5) |
| Cardiac failure chronic | 1 (0.5) |
| Myocardial infarction | 1 (0.5) |
| Gastrointestinal disorders | 3 (1.4) |
| Large intestine polyp | 2 (1.0) |
| Diarrhoea | 1 (0.5) |
| Inguinal hernia | 1 (0.5) |
| General disorders and administration site conditions | 1 (0.5) |
| Hernia | 1 (0.5) |
| Infections and infestations | 2 (1.0) |
| Gangrene | 1 (0.5) |
| Periodontitis | 1 (0.5) |
| Injury, poisoning and procedural complications | 3 (1.4) |
| Heat stroke | 1 (0.5) |
| Patella fracture | 1 (0.5) |
| Tibia fracture | 1 (0.5) |
| Metabolism and nutrition disorders | 2 (1.0) |
| Diabetes mellitus | 1 (0.5) |
| Hypoglycaemia | 1 (0.5) |
| Neoplasms benign, malignant, and unspecified (incl cysts and polyps) | 2 (1.0) |
| Gastric cancer | 1 (0.5) |
| Prostate cancer | 1 (0.5) |

| | |
|---|---|
| Nervous system disorders | 3 (1.4) |
| Carpal tunnel syndrome | 1 (0.5) |
| Cerebellar infarction | 1 (0.5) |
| Cerebral infarction | 1 (0.5) |
| Psychiatric disorders | 1 (0.5) |
| Suicidal behaviour | 1 (0.5) |
| Renal and urinary disorders | 7 (3.3) |
| Diabetic nephropathy | 3 (1.4) |
| Chronic kidney disease | 2 (1.0) |
| End stage renal disease | 1 (0.5) |
| Renal failure | 1 (0.5) |
| Respiratory, thoracic and mediastinal disorders | 1 (0.5) |
| Interstitial lung disease | 1 (0.5) |

If a patient had multiple SAEs within a primary system organ class, the event was only counted once for that system organ class. If a patient had multiple SAEs of a preferred term, the event was only counted once for that preferred term.

Data are classified according to the System Organ Class and Preferred Terms of MedDRA version 22.0.

MedDRA, medical dictionary for regulatory activities; SAE, serious adverse event.
