# Supplementary material for: Impact on visual acuity and psychological outcomes of ranibizumab and subsequent treatment for diabetic macular oedema in Japan (MERCURY)
Source: Graefes Arch Clin Exp Ophthalmol. 2021 Sep 3;260(2):477–87. doi: 10.1007/s00417-021-05308-8 (PMC8786783; doi:10.1007/s00417-021-05308-8)
Supplement: Supplementary file 14 — Supplementary file14 (PDF 207 KB) [file 417_2021_5308_MOESM14_ESM.pdf]

Graefe's Archive for Clinical and Experimental Ophthalmology

Impact on visual acuity and psychological outcomes of ranibizumab and subsequent treatment for diabetic macular oedema in Japan (MERCURY)

Taiji Sakamoto, Masahiko Shimura, Shigehiko Kitano, Masahito Ohji, Yuichiro Ogura, Hidetoshi Yamashita, Makoto Suzaki, Kimie Mori, Yohei Ohashi, Poh Sin Yap, Takeumi Kaneko, Tatsuro Ishibashi, for the MERCURY Study Group

Corresponding author:

Taiji Sakamoto

Department of Ophthalmology, Kagoshima University, 8-35-1 Sakuragaoka, Kagoshima 890-8544, Japan

Tel: +81 99-275-5402

Fax: +81 99-265-4894

Email: tsakamot@m3.kufm.kagoshima-u.ac.jp

Online Resource 14. Visual acuity gain during anti-VEGF therapy for diabetic macular oedema: Real-world data

Study	Patients, *n*	Eyes, *n*	Follow-up, months	Treatment regimen	Anti-VEGF injections at month 12, mean ± SD	Mean baseline BCVA (ETDRS)	Mean change in BCVA (ETDRS) from baseline to month 12
MERCURY	209	209[a]	12	ND	3.6 ± 2.4	63.5	4
Moorfields DME Patrao et al. 2016 [25]	164	200	12	3+PRN	7.2 ± 2.3	54.4	6.6
Epstein et al. 2018 [30]	80	102	48	3+PRN	7.7 ± 3.4[b]	60.8	6.6[b]
Holekamp et al. 2018 [26]	110	121	12	ND	3.1 ± 2.4	56.9	4.7
OCEAN Ziemssen et al.	1,226	-	12	ND	4.4	60.6	4

2018 [27]							
STREAT-DME Shimura et al. 2020 [31]	1,552	2,049	24	ND	3.8 ± 3.3[c]	63	2[c]
BOREAL-DME Massin et al. 2019 [28]	290	290	12	ND	5.1 ± 2.3	59.2	7.4
LUMINOUS Mitchell et al. 2020 [29]	1,049	-	12	ND	4.5 ± 2.5	57.7	3.5

[a]Primary treated eyes. [b]4-year data. [c]2-year data.

BCVA, best-corrected visual acuity; ETDRS, early treatment diabetic retinopathy study; ND, not determined; PRN, *pro re nata*; SD, standard deviation; VEGF, vascular endothelial growth factor.
